# Determinants of Fatigue after First-Ever Ischemic Stroke during Acute Phase

**DOI:** 10.1371/journal.pone.0110037

**Published:** 2014-10-10

**Authors:** Shan-Shan Wang, Jia-Ji Wang, Pei-Xi Wang, Ruoling Chen

**Affiliations:** 1 Institute of Public Health, School of Nursing, Henan University, Kaifeng, China; 2 Department of Preventive Medicine, School of Public Health, Guangzhou Medical University, Guangzhou, China; 3 Department of Primary Care and Public Health Sciences, King’s College London, London, United Kingdom; 4 Centre for Health and Social Care Improvement (CHSCI), University of Wolverhampton, Wolverhampton, United Kingdom; University of Glasgow, United Kingdom

## Abstract

**Background and Purpose:**

Fatigue after stroke is common and has a negative impact on rehabilitation and survival. However, its pathogenesis and contributing factors remain unclear. The purpose of this study was to identify factors influencing the occurrence of fatigue after first-ever ischemic stroke in acute phase.

**Methods:**

We examined 265 consecutive patients with first-ever ischemic stroke during acute phase (within 2 weeks) in two tertiary stroke care hospitals in Henan, China. We documented patients’ demographic and clinical characteristics through face-to-face interviews using structured questionnaires and reviews of medical records. Post-stroke fatigue was defined as a score of ≥4 using the Fatigue Severity Scale. Multivariate logistic regression was used to examine post-stroke fatigue in relation to socio-demographic, lifestyle, clinical characteristics and family function.

**Results:**

About 40% first-ever ischemic stroke patients experienced post-stroke fatigue in acute phase. Post-stroke fatigue was associated with lack of exercise before stroke (adjusted odds ratio 4.01, 95% CI 1.95–8.24), family dysfunction (2.63, 1.20–5.80), depression (2.39, 1.02–5.58), the presence of pre-stroke fatigue (4.89, 2.13–11.21), use of sedative medications (4.14, 1.58–10.88), coronary heart disease (3.38, 1.46–7.79) and more severe Modified Rankin Scale (2.55, 1.65–3.95).

**Conclusions:**

The causes of post-stroke fatigue are multifaceted. More physical exercise, improving family function, reducing depression and appropriate use of sedative medications may be helpful in preventing post-stroke fatigue.

## Introduction

In recent years, post-stroke fatigue (PSF), one of the most common, enduring and disabling complaints after stroke, has been increasingly recognized [1]–[3]. Fatigue is multidimensional and comprises physical, emotional and cognitive experiences [4]. Although the definition of PSF is still a subject of controversy, it is generally described as a sense of early exhaustion with weariness, lack of energy and aversion to effort [5] that develops during physical or mental activity and is usually not ameliorated by rest [6]. The prevalence of PSF varies between 23% and 75% [7]. PSF has a negative impact on stroke survivors’ rehabilitation, neurological recovery, quality of life and work capacity [8], [9]. More seriously, PSF increases the risk of suicide [10] and all-cause mortality [11]. The consequences of fatigue should not be underestimated. It is considered by many stroke patients as one of the most troublesome symptoms to deal with [5].

Previous studies showed that potential predisposing factors for PSF could be older age, female gender, neurological deficits, complications, use of medications, sleep disturbances, pre-stroke fatigue, depression, cognitive ability decline and lesion location [7]. However, PSF has also been reported in patients who are not depressed, and have little neurological or motor impairment [12]–[13]. It is uncertain whether other factors, such as exercise before stroke and family function, may affect the risk of PSF.

For most patients, PSF develops from the acute stage of stroke. In some studies on PSF in acute phase in all types of stroke, the incidence of PSF has been reported to be 25%–70% [3], [14]–[18]. More importantly, the acute phase following stroke may represent a critical period for rehabilitation [19]. It is therefore necessary to identify possible risk factors for PSF in acute phase for developing effective prevention measures. Ischemic stroke accounts for the vast majority of stroke patients, and the proportion has increased over time globally. To the best of our knowledge, there has been no research on the determinants of PSF in patients with first-ever ischemic stroke in acute phase. This study was aimed to estimate the prevalence of fatigue after first-ever ischemic stroke during acute phase and examine its determinants.

## Methods

### Participants and Procedures

This was a cross-sectional observational study. With an estimated post-stroke fatigue (PSF) frequency of about 40% in study hospitals, at an alpha error of 5% and a power of 80%, a sample size of 266 was required to detect a dichotomous risk factor with odds ratio (OR) of 2.0 or greater in association with PSF. All consecutive patients with first-ever ischemic stroke during acute phase (the first 2 weeks following a stroke) [15] were recruited upon informed consented between July 1, 2013 and December 31, 2013 at the neurology department in Huai-He Hospital and The First Hospital Affiliated to Henan University, Kaifeng, China. Patients in the intensive care unit were excluded. All patients received conservative treatment. No patients received thrombolytic treatment due to delays in arrival for initiating effective thrombolytic treatment or worries about serious complications of thrombolytic treatment. Data were collected in standardized interviews using validated questionnaires and through reviews of medical records. Patient’s demographic and clinical characteristics were recorded on admission. Before the interview, participants were informed that one of their main caregivers could take part in the interview. The interview could be conducted in the patient’s in-hospital room or other quiet place.

**Figure 1:**
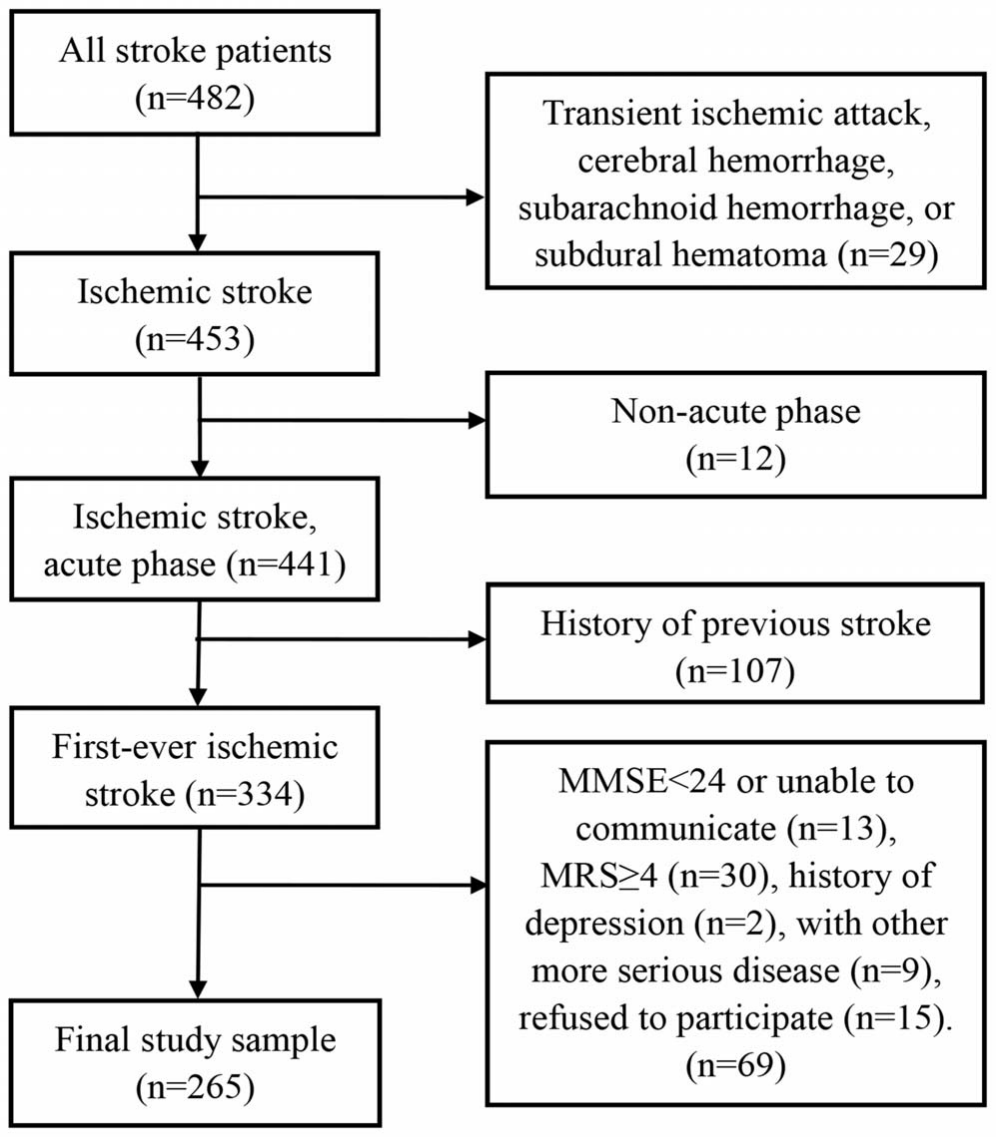
Flow chart in the selection of study patients.

During the study period, we recruited 265 consecutive participants with first-ever ischemic stroke (sudden loss of blood circulation to an area of brain resulting in a corresponding loss of neurologic function) according to the diagnostic criteria of the fourth Chinese national conference’s recommendations on the diagnosis of cerebrovascular diseases [20]. All diagnoses were confirmed by CT or MRI. The inclusion criteria were as follows: (1) age of 18 years or older, (2) stable condition and conscious, or somnolent but able to answer questions, (3) sufficient cognitive (MMSE score more than 24) and speech function to participate, or with language impairment but written ability to complete the study questionnaires, (4) Modified Rankin Scale (MRS) <4, because patients with MRS ≥4 were unable to move by themselves and some items of the fatigue severity scale were not applicable, and (5) willingness to provide the informed consent. Patients were excluded if they: (1) were transient ischemic attack, cerebral hemorrhage, subarachnoid hemorrhage, or subdural hematoma; (2) had a previous history of stroke; (3) had more serious medical disease other than ischemic stroke, such as cancer, renal failure, or Parkinson’s disease; (4) had a previous history of depression diagnosed by a medical doctor. A flowchart illustrating the selection of study patients is presented in Figure 1.

### Measurements

On admission after informed consent, trained research staff collected data on socio-demographic, lifestyle and clinical characteristics. Depressive symptoms, sleep disturbances, sedative medications and fatigue after stroke were assessed at 13–14 days after stroke before discharge in stable clinical conditions (the routine in-hospital treatment course for stroke is 14 days).

#### Socio-demographic and lifestyle variables

Demographic information included age, gender, marital status, place of residence (urban or rural), education, family income (<2000 yuan/month or ≥2000 yuan/month), lifestyle before stroke (including smoking, alcohol drinking and exercise) and body mass index (BMI). Education level was categorized into ≤9 years (middle school), 10–12 years (high school), and ≥13 years (university). Exercise before stroke was defined as strenuous if lasting longer than 30 minutes, three times a week, and moderate if lasting less than 30 minutes, or none if there was no exercise [21]. Patients’ weight and height were measured on admission, and BMI was categorized according to Chinese weight criteria for adults.

#### Possible stroke-related clinical risk factors

*General Assessment*. On admission, stroke severity was assessed using the National Institute of Health Stroke Scale (NIHSS) [22]. Modified Rankin Scale (MRS) [23], which rating the dependency of patients ranging from 0 (no symptom) to 5 (severe disability), was assessed after in-hospital treatment in stable clinical conditions before discharge. According to NIHSS score, participants were categorized into minor ischemic stroke (NIHSS≤3) and moderate to severe ischemic stroke (NIHSS≥4) [24]. According to MRS, patients were classified as favorable (MRS 0–1) or unfavorable (MRS ≥2) in clinical prognosis [18]. Location of stroke was obtained from medical records and was categorized as left, right, or bilateral based on CT or MRI. Comorbidities included hypertension, coronary heart disease, diabetes and hyperlipidemia diagnosed by a physician. Pre-stroke fatigue [15] was measured by two questions: “did you experience fatigue before you had your stroke” (yes/no), and if yes, “how long did you experience fatigue” (less than a week, less than 3 months, more than 3 months). Patients who chose fatigue lasting longer than 3 months before the stroke were defined as having pre-stroke fatigue. In addition, patients were asked about sleep disturbances (i.e., insomnia or frequent awakening during nighttime sleep) and use of sedative and sleeping pills (all benzodiazepines in study hospitals).

*Depressive Symptoms*. Depression after stroke was evaluated using Beck Depression Inventory Version II (BDI-II) [25]. Previous studies showed that BDI-II is an acceptable instrument for depression [26] and has satisfactory validity and reliability [15]–[16] in stroke patients. The scale consists of 21 items, each scores 0–3. Total score ranges from 0 to 63. The best possible score is 0. A score cut-off value of 13 was used to categorize participants as not depressed (total score ≤13) or depressed (total score ≥14) [26]. Higher scores indicate more serious depression.

*Family function*. Personal health, the occurrence and recovery of disease may be closely linked to family function and resources. In this study, personal satisfaction with family function was measured by Family APGAR Index [27]–[28]. This scale is composed of 5 elements: adaptation, partnership, growth, affection and resolve. Participants chose according to the frequency of feeling satisfied with each of the five items using a 3-point scale ranging from 0 (hardly ever) to 2 (almost always). Total score ranges from 0 to 10. A score of 0–3 indicates severe family dysfunction, 4–7 moderate family dysfunction, and 8–10 functional family. Based on previous studies, Cronbach’s alpha coefficient was 0.80 to 0.84 [27]–[28].

#### Post-Stroke Fatigue

PSF was assessed by Fatigue Severity Scale (FSS). FSS reflects the influence of fatigue on daily life, and is one of the most frequently used fatigue instruments in stroke studies [29]–[30]. It has good validity and reliability [30]–[31]. The scale contains nine statements, each of them is scored on a 7-point Likert scale, ranging from 1 (completely disagree) to 7 (completely agree). It is relatively fast and simple to understand and complete. The average score of the nine items was computed, representing the overall total FSS score for each patient. Patients with a total score of ≥4 points are classified as “fatigued”; higher scores indicate more severe fatigue [32]. Recently, a study of PSF in acute phase showed that the internal consistency of FSS was adequate (Cronbach’s α coefficients = 0.85) [15]. Cronbach’s alpha coefficient of FSS in the present study was 0.94.

### Ethics statement

This study was approved by the research ethics committee of Henan University. Written informed consent was obtained from all study participants.

### Data analysis

We examined differences in characteristics between patients with and without PSF, using T-test for continuous variables and χ^2^ test for categorical variables. Multivariate logistic regression models were employed to investigate the risk factors of PSF. Parsimonious final logistic regression model was fitted by retaining all predictor variables with P<0.05. Odds ratios (OR) with 95% confidence intervals are presented. There was no significant co-linearity between predictor variables affecting the stability of the regression models. Two tailed p values <0.05 were considered statistically significant. All analyses were conducted using Statistical Package for Social Sciences (SPSS) 13.0 (SPSS, Inc, Chicago IL).

## Results

### Participant characteristics

A summary of characteristics in study patients are presented in **Table 1**. In total, 265 patients with first-ever ischemic stroke in acute phase were included. Patients aged between 30 to 95 years, with an average of 63.0 years (SD = 12.1). Male patients comprised 57% of the study sample. The majority of participants were married (86.8%), lived in urban area (57.7%), had education less than 9 years (69.4%) and had a family income of more than 2000 RMB/month (73.6%). The proportions of smoking, alcohol drinking and exercise before stroke were 32.1%, 30.6% and 71.3% respectively. 73.6% of patients were from a functional family.

**Table 1:** Characteristics of ischemic stroke participants with and without post-stroke fatigue.

	Fatigue	No fatigue	
Variable	(n = 106)	(n = 159)	*p value*
Age, mean(SD)	63.2(12.1)	62.8(12.1)	0.810
Gender			0.076
Male	53(50.0)	98(61.6)	
Female	53(50.0)	61(38.4)	
Marital status			0.465
Single	16(15.1)	19(11.9)	
Married	90(84.9)	140(88.1)	
Residence			0.043
Urban	53(50.0)	100(62.9)	
Rural	53(50.0)	59(37.1)	
Education			0.903
≥13 years	12(11.3)	18(11.3)	
10–12 years	19(17.9)	32(20.1)	
≤9 years	75(70.8)	109(68.6)	
Family income			0.003
≥2000 RMB/month	67(63.2)	128(80.5)	
<2000 RMB/month	39(36.8)	31(19.5)	
BMI			0.483
Underweight(<18.5)	4(3.8)	8(5.0)	
Normal(18.5–23.9)	45(42.5)	61(38.4)	
Overweight(24.0–27.9)	46(43.4)	65(40.9)	
Obesity(≥28.0)	11(10.4)	25(15.7)	
Life styles before stroke			
Smoking			0.227
Yes	29(27.4)	56(35.2)	
No	77(72.6)	103(64.8)	
Alcohol drinking			0.174
Yes	27(25.5)	54(34.0)	
No	79(74.5)	105(66.0)	
Exercise before stroke			<0.001
Yes	60(56.6)	129(81.1)	
No	46(43.4)	30(18.9)	
Family function			<0.001
Functional	61(57.5)	134(84.3)	
Moderate/severe dysfunction	45(42.5)	25(15.7)	

Data presented are n (%) or mean (SD); Fatigue defined as Fatigue Severity Scale score ≥4; BMI, Body mass index; Family function: Family APGAR index.

### The prevalence of fatigue and related variables

The average FSS score was 3.5±1.6. During the acute phase after first-ever ischemic stroke, 159 (60.0%, 95% CI 54.1%–65.9%) experienced no fatigue (FSS<4) and 106 (40.0%, 95% CI 34.1%–45.9%) complained fatigue (FSS≥4).

To identify demographic, lifestyle and clinical factors associated with fatigue after acute first-ever ischemic stroke, we first compared patients with versus without PSF in bivariate analyses (**Tables 1** **and** **2**). PSF patients were more likely to live in rural area, and to have low family income, lack of exercise before stroke, or family dysfunction. There were no significant differences in age, gender, marital status, education, BMI, smoking and alcohol drinking between the 2 groups. PSF was significantly associated with pre-stroke fatigue, coronary heart disease, depression, sleep disturbances, use of sedative medications, MRS and NIHSS score, but was not related to hypertension, diabetes, hyperlipidemia and location of the ischemic lesion.

**Table 2:** Clinical factors related to post-stroke fatigue in ischemic stroke participants.

	Fatigue	No fatigue	
Variablez	(n = 106)	(n = 159)	*p value*
Comorbidity			
Hypertension	59(55.7)	97(61.0)	0.445
Diabetes	33(31.1)	37(23.3)	0.159
Coronary heart disease	34(32.1)	17(10.7)	<0.001
Hyperlipidemia	45(42.5)	64(40.3)	0.799
Pre-stroke fatigue	33(31.1)	16(10.1)	<0.001
Depression	47(44.3)	15(9.4)	<0.001
Sedative medications	23(21.7)	11(6.9)	0.001
Sleep disturbances	44(41.5)	33(20.8)	<0.001
MRS			<0.001
0–1	14(13.2)	75(47.2)	
2–3	92(86.8)	84(52.8)	
NIHSS score			<0.001
≤ 3	45(42.5)	104(65.4)	
≥ 4	61(57.5)	55(34.6)	
Location			0.614
Left	40(37.7)	66(41.5)	
Right	43(40.6)	55(34.6)	
Bilateral	23(21.7)	38(23.9)	

Data presented are n(%); Fatigue defined as Fatigue Severity Scale score ≥4; Depression, the Beck Depression Inventory Version II (BDI-II); MRS, Modified Rankin Scale; NIHSS, National Institute of Health Stroke Scale.

A number of independent predictors of PSF after first-ever ischemic stroke were revealed in multivariate logistic regression analyses (**Table 3**). After the multivariate adjustment, fatigue after first-ever ischemic stroke was significantly related to severe MRS (OR = 2.55, 95% CI 1.65–3.95), depressive symptoms (OR = 2.39, 95% CI 1.02–5.58), lack of exercise before stroke (OR = 4.01, 95% CI 1.95–8.24), the presence of pre-stroke fatigue (OR = 4.89, 95% CI 2.13–11.21), use of sedative medications (OR = 4.14, 95% CI 1.58–10.88), having coronary heart disease (OR = 3.38, 95% CI 1.46–7.79) and family dysfunction (OR = 2.63, 95% CI 1.20–5.80). The associations with rural living, low family income, sleep disturbances and NIHSS score were not statistically significant (all P>0.2) and not included in the final parsimonious regression model.

**Table 3:** Factors related to post-stroke fatigue from multivariate logistic regression models.

Factor	*Odds Ratio*	*95%CI*	*P*
MRS			
0–1	referent		
2–3	2.55	1.65–3.95	<0.001
Depression			
No	referent		
Yes	2.39	1.02–5.58	0.04
Exercise before stroke			
Yes	referent		
No	4.01	1.95–8.24	<0.001
Pre-stroke fatigue			
No	referent		
Yes	4.89	2.13–11.21	<0.001
Sedative medications			
No	referent		
Yes	4.14	1.58–10.88	0.004
Coronary heart disease			
No	referent		
Yes	3.38	1.46–7.79	0.004
Family funtion			
Highly functional family	referent		
Moderate/severe family dysfunction	2.63	1.20–5.80	0.02

Post-stroke fatigue defined as Fatigue Severity Scale score ≥4 at 13–14 days after stroke; OR = Odds ratio; CI = confidence interval; MRS, Modified Rankin scale.

## Discussion

### Main findings

In this Chinese study population, our results confirmed several risk factors of PSF as reported in previous studies. In addition, we found that post-stroke fatigue was associated with lack of exercise before stroke and family dysfunction.

### Comparisons with previous studies

In our study, 40% of first-ever ischemic stroke patients experienced post-stroke fatigue during acute phase. This is not significantly different from the findings in previous studies of patients with all types of stroke in acute phase. For example, using the same instrument, a study in Dutch rehabilitation centers reported that 51.5% patients in acute stage of a first-ever stroke experienced fatigue [17]. Another study from Norway showed that 57% of patients experienced fatigue after stroke during acute phase [15]. High prevalence of PSF requires more attention on its risk factors for initiating preventive measures.

In recent years, many investigators have demonstrated that depressive symptoms and pre-stroke fatigue are independent predictors of PSF [1], [11], [15]–[16], [18], [33]–[34]. Similarly, we found that depressive symptoms and pre-stroke fatigue were related to PSF in acute phase even after adjustment for other significant co-variables. A recently published longitudinal study also reported that pre-stroke fatigue was independently associated with an increased risk of fatigue in the post-stroke period [16]. Reducing depression and fatigue may be helpful for preventing PSF.

In this study, we found a significant association between MRS and PSF in acute phase. This is consistent with previous studies reporting severe MRS as an independent risk factor for PSF [1], [13]. However, a study in the Netherlands showed no association between MRS and PSF [18]. The main reasons for the lack of association in their study may be that patients with severe neurological conditions (MRS score ≥4) were included, and that their sample size was relatively small (n = 108). The MRS represents the degree of disability which is one of the external manifestations of neurological deficits. MRS may be an important predictor of fatigue after first-ever ischemic stroke.

The use of medications was another factor independently associated with PSF in acute phase. Similar findings had been reported in some [11], [34] but not other [15] previous studies. The differential findings might be due to different types of drugs. In our study, medications included sedative and sleeping pills which can produce malaise and drowsiness, so patients may experience fatigue. Although most previous studies have not considered medications as an independent risk factor of PSF, our study findings suggest the need to clarify the relationships between various medications and PSF.

On the relationships between comorbidities and PSF, the findings have been inconsistent in previous studies. Naess et al. indicated a clear association between the presence of coronary heart disease and PSF [11]. This is consistent with our study findings. In contrast, Appelros et al. reported no relationship between comorbidities and PSF [13], [34]. This may be partly related to the differential timing in evaluating the PSF. PSF was assessed at 13–14 days after stroke in our study, but at least 3 months after stroke in Appelros et al’s study. Fatigue is a common symptom of heart disease. Our data suggest that having coronary heart disease may be a potential predisposing factor for PSF in acute phase.

An important new finding from our study is the significant association between lack of exercise before stroke and PSF in acute phase. Exercise can improve physical fitness, thereby may reduce fatigue. Thus, this finding is not a surprise. Regular exercise may be helpful in reducing PSF, apart from preventing stroke.

Another new finding we uncovered is that family dysfunction may be a potential risk factor for PSF. Personal health, the occurrence and recovery of disease are closely linked to family function. Family dysfunction can in turn result in a range of psychological and behavioral problems, such as drug abuse and depression [35]. Medications and depression have been shown to be independent predictors of PSF. Improving family function may be helpful in preventing PSF.

### Strengths and weaknesses of the study

In this study, we used standardized questionnaires in collecting many variables on patients’ socio-demographic and clinical characteristics. This provided a unique opportunity to examine the determinants of PSF. However, our study included a relatively small number of patients, and the 95% confident intervals of estimated odds ratios for PSF are relatively wide. Our study excluded patients with more severe cognitive impairment and MRS ≥4; the findings might not be generalizable to all ischemic stroke patients. Our study design is cross-sectional, and causal relationships could not be firmly established. We collected data on pre-stroke fatigue retrospectively, information recall bias might exist. Large, prospective cohort studies are required to confirm the observed associations, particularly on the impact of exercise before stroke and family function.

## Conclusions

Fatigue after stroke in acute phase was associated with lack of exercise before stroke, family dysfunction, depressive symptoms, the presence of pre-stroke fatigue, use of sedative medications, coronary heart disease and severe MRS. These findings suggest measures for preventing PSF. These may include promoting regular physical exercise, improving family function, timely treatment of complications, rational use of sedative medications and psychological counseling to ease negative emotions.
