# Correction: Determinants of Fatigue after First-Ever Ischemic Stroke during Acute Phase

**DOI:** 10.1371/journal.pone.0116461

**Published:** 2014-12-19

**Authors:** The PLOS ONE Staff

The affiliation for the fourth author is incorrect. Ruoling Chen is not affiliated with #3 but with #4 Centre for Health and Social Care Improvement (CHSCI), University of Wolverhampton, Wolverhampton, United Kingdom.

## References

[1] Wang S-S, Wang J-J, Wang P-X, Chen R (2014) Determinants of Fatigue after First-Ever Ischemic Stroke during Acute Phase. PLoS ONE 9(10): e110037 doi:10.1371/journal.pone.0110037 25302807 10.1371/journal.pone.0110037 PMC4193856

